# Reply to Cione et al. Comment on “Iida et al. Development of a New Method for Calculating Intraocular Lens Power after Myopic Laser In Situ Keratomileusis by Combining the Anterior–Posterior Ratio of the Corneal Radius of the Curvature with the Double-K Method. *J. Clin. Med.* 2022, *11*, 522”

**DOI:** 10.3390/jcm11102708

**Published:** 2022-05-11

**Authors:** Yoshihiko Iida, Kimiya Shimizu, Nobuyuki Shoji

**Affiliations:** 1 Department of Ophthalmology, Kitasato University School of Medicine, Sagamihara 252-0374, Japan; nshoji@kitasato-u.ac.jp; 2 Eye Center, Sanno Hospital, Tokyo 107-0052, Japan; kimiyas@iuhw.ac.jp

We appreciate the insightful comments [1] on our article [2]. We reply as follows:

(1) We agree with their opinion that the paper by Rosa et al., where a formula to estimate keratometry (K) before refractive surgery (Kpre) based on the postoperative posterior corneal power was proposed [3], is important for this topic and thus should have been cited in this article.

(2) The corneal refractive power used in the SRK/T formula is optimized to the keratometric value, which estimates the total corneal refractive power from the anterior surface of the cornea. The ISS method is a double-K method based on the SRK/T formula using the K value with the IOL master and the axial length of the eye, and it is not appropriate to directly input the Pentacam measurements as the Kpost [4]. Therefore, the ISS method uses the IOL Master measurements to calculate IOL power using the Double-K method, determines the C-factor based on the correlation between the refractive error of Double-K method and the A-P ratio derived from Pentacam, and adjusts the target refraction value. The Pentacam measurements are only used to determine the A-P ratio of corneal curvature, and do not correspond to the point made by Cione et al. that data derived from different machines cannot be used interchangeably. Incidentally, there are other instruments besides the Pentacam that can measure and calculate the anterior–posterior corneal curvature ratio, including anterior segment OCT, but since the measurement principle is different, we believe that the anterior–posterior corneal curvature ratio measured with anterior segment OCT cannot be used in the ISS method without modification. Therefore, it should be noted that the calculation of the A-P ratio is not compatible between different models. If the A-P ratio measured by anterior segment OCT is to be used, it is necessary to calculate the C-factor from the correlation between the A-P ratio measured by anterior segment OCT and the refractive error of the Double-K method.

(3) Our study was designed to compare the ISS method with other IOL calculation methods commonly used for cataract surgery in the post-LASIK eye, primarily those included in the American Society of Cataract and Refractive Surgery calculator, rather than an exhaustive comparison of all methods. We hope to compare other IOL power calculations in the future, including ALMA [5], which is not included in the ASCRS calculator.

(4) Regarding the evaluation of PE, as has been pointed out, the conversion from IOL PE to PE is an estimate, and there may be differences in the amount of PE change per 1 D depending on the IOL degree. In the present study, the mean IOL power was 20.63 ± 2.20 D (15.0–24.0 D), and many cases used a power around 20.0 D, which may have caused errors in the IOL PE to PE conversion but had relatively little effect on the results. Furthermore, the optical design of IOLs differs depending on the type of IOL, which may affect the amount of change, but only one type of IOL was used in this study, and the influence of the type of IOL on the error was eliminated. However, this is a limitation of this evaluation method, as indicated by Cione et al.

(5) Although the IOL power calculation analysis for normal eyes requires a process of optimizing the IOL constant for the data set to achieve a mean error (ME) of 0, deriving an optimized IOL constant for a special case such as post-LASIK eyes in clinical practice is not an exact process or applicable to future cases; some have suggested that it is inappropriate because it is not considered possible [6]. In addition, the number of cases of atypical eyes is small, and it is difficult to optimize the IOL constants. What is clinically required is a formula that can be used with the same constants as for normal eyes and still provide accurate results. Therefore, in this study, IOL power calculations were performed using standard IOL constants optimized for normal eyes, and no further optimization of the data set of IOL constants was performed. The ISS method has the advantage of using the A constant of the SRK/T formula, which is optimized for normal eyes.

(6) The papers published in the past 10 years indicate that it is advisable to publish studies that include a sample size of about 200 eyes for normal eyes and at least 50 eyes for atypical eyes, such as after refractive surgery [7]. The cases in the current study were limited to one eye per patient and one type of IOL, which we hope will reduce the possibility of statistical error. It should be added that all of the objective eyes in this study were limited to cases in which 20/20 or better visual acuity was achieved, which does not mean that the accuracy of the postoperative refractive error is insufficient.

As for the statistics, data were verified for non-normality using the Kolmogorov–Smirnov test. The percentages of eyes within ±0.25 D, ±0.50 D, and ±1.00 D of PE were compared using Fisher’s exact test and Bonferroni correction, following the methods used in previous reports [8,9].

We compared the percentage of eyes within ±0.25, ±0.50 and ±1.00 D of PE by Cochran’s Q test [7], as suggested by Cione et al. The percentages of eyes within ±0.25 D, ±0.50 D, and ±1.00 D of PE were significantly different (*p* < 0.0001, *p* < 0.0001, *p* = 0.006), respectively. Furthermore, the comparison between formulas was analyzed by the McNemar test with Bonferroni correction, and the ISS method was significantly better than the Potvin–Hill Pentacam method at ±0.25 D (*p* = 0.015). At ±0.50 D, the ISS method was better than the Potvin–Hill Pentacam method (*p* = 0.044) and significantly better than the Haigis-L formula (*p* = 0.044), and at ±1.00 D, the Cochran Q test showed a significant difference, but no significant difference could be detected in the comparison between each formula.

We would like to add a comment about the evaluation of the median absolute value of error (MedAE), as suggested by Cione et al. The Friedman test with a post hoc test (Bonferroni multiple test) showed that the ISS method was significantly different from all formulas (*p* < 0.0001 for all) except the Barrett True-K formula; the Shammas No-history method and the Haigis-L formula were significantly different from the Potvin–Hill Pentacam method (*p* = 0.001, *p* = 0.0001); and the Barrett True-K formula was significantly different from the Shammas No-history method and the Potvin-Hill Pentacam method (*p* = 0.019, *p* < 0.0001).

As mentioned above, although results with additional statistical analysis are also shown, the predictability of the ISS method was equal to or better than several other formulas. In addition, it should be noted that Pentacam should be used when using the A-P ratio of corneal curvature radius in the ISS method, and that the A-P ratio is not compatible with other instruments. We thank the authors again for their commentary on the evaluation methods of the multiple IOL frequency formulas used in this study.

## Data Availability

The data presented in this study are available on request from the corresponding author.
